# Endoscopic management of colocolonic anastomotic dehiscence: a case report

**DOI:** 10.1016/j.igie.2026.02.013

**Published:** 2026-02-23

**Authors:** Inês Rodrigues Simão, Rui Mendo, Pedro C. Figueiredo

**Affiliations:** Gastroenterology Department, Unidade Local de Saúde Lisboa Ocidental, Hospital de Egas Moniz, Lisboa, Portugal

## Abstract

We describe the case of a woman who developed a perianastomotic collection after colocolonic anastomosis for restoration of bowel continuity, consistent with an anastomotic dehiscence. Endoscopy revealed a stenotic anastomosis inaccessible to vacuum therapy. A fully endoscopic strategy was pursued, combining internal drainage of the collection with double-pigtail stents and treatment of the stenosis through balloon dilation. The patient showed a favorable course, with complete resolution on follow-up. This case underscores the feasibility, safety, and efficacy of endoscopic management of colocolonic anastomotic dehiscence, highlighting the importance of simultaneously addressing associated stenosis.

## Introduction

Anastomotic dehiscence after colorectal surgery remains a serious adverse event, with an incidence ranging from 1.5% to 19% and substantial morbidity and mortality.^1^, ^2^, ^3^, ^4^ Management strategies are tailored to the clinical presentation. Nonsurgical options are favored for contained leaks, such as antibiotic therapy, percutaneous or transanal drainage, whereas surgery is reserved for generalized peritonitis or failed conservative treatment.^1^^,^^5^, ^6^, ^7^

Endoscopic therapy has emerged as a minimally invasive alternative for managing anastomotic dehiscence. Reported modalities include endoscopic vacuum therapy (EVT), self-expandable metal stents, over-the-scope clips, and internal drainage with double-pigtail stents. Robust evidence supports EVT as a highly effective treatment for colorectal leaks.^8^, ^9^, ^10^ However, most studies focus on rectal anastomoses, with only limited data addressing colocolonic dehiscence.^11^

## Case report

A 70-year-old woman with hypertension, type 2 diabetes, and dyslipidemia had a history of iatrogenic rectal perforation after an enema for constipation treatment, initially managed with diverting colostomy. Two years later, she underwent colonic transit reconstruction with a mechanical colocolonic anastomosis. One month postoperatively, she presented to the emergency department with a 24-hour history of generalized abdominal pain and vomiting. She was hemodynamically stable, with a temperature of 37.8 °C. Abdominal examination revealed tenderness to palpation without guarding or rebound tenderness. Laboratory studies revealed elevated inflammatory markers, with leukocytosis of 20.8 × 10^9^/L (normal range, 4.0-10.0) and a C-reactive protein of 26.9 mg/dL (normal <0.5). Abdominal computed tomography scan showed an 80-mm perianastomotic collection consistent with dehiscence (Fig. 1). She was started on intravenous antibiotics and kept nil per os with parenteral nutrition.

**Figure 1:**
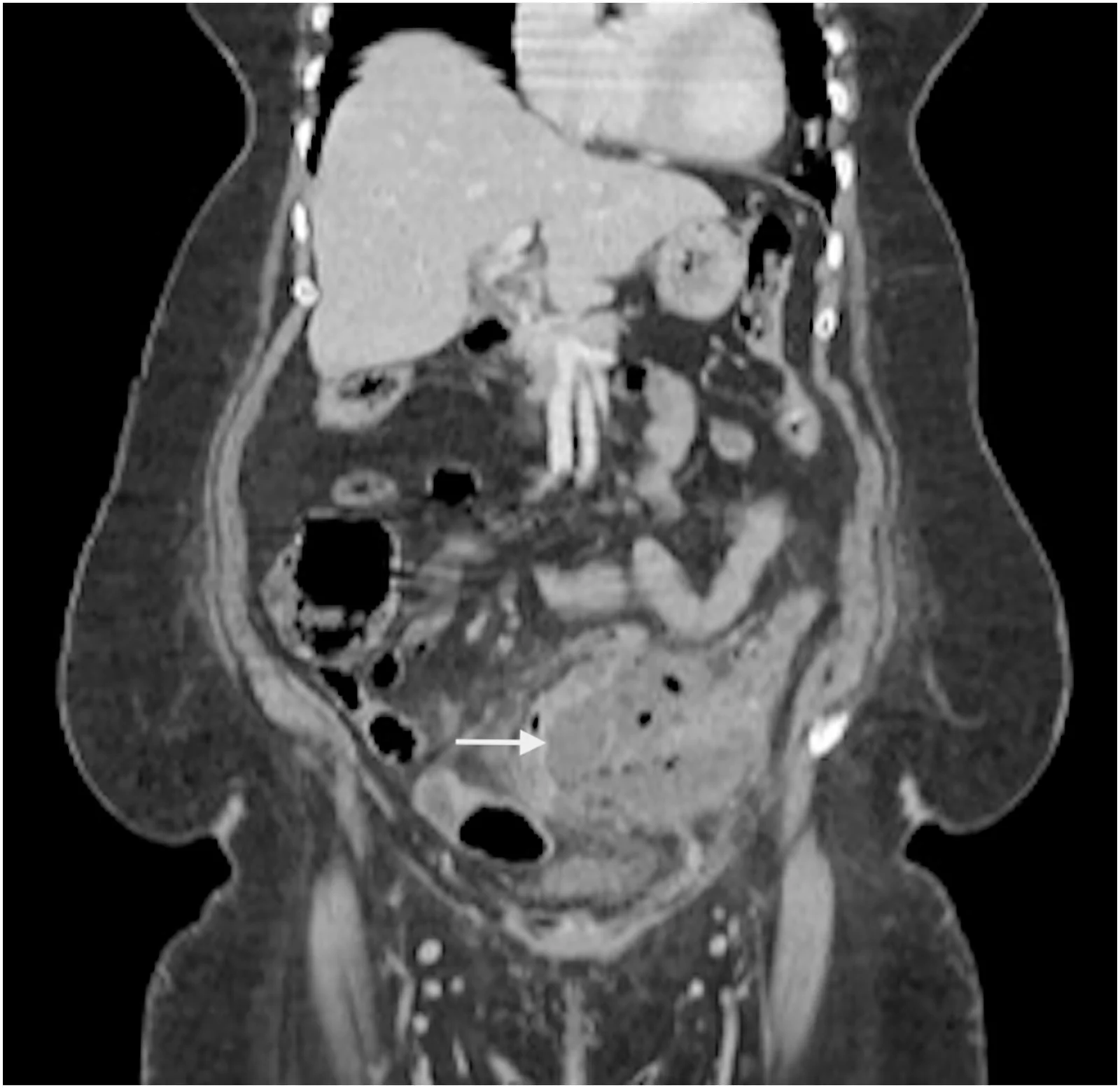
Initial computed tomography scan showing an 8-cm collection (*arrow*).

The case was discussed in a multidisciplinary meeting involving the surgical and gastroenterology teams, and the patient was proposed for EVT. Colonoscopy, performed on the third day of admission, revealed a 10-mm-wide inflammatory anastomotic stenosis, 30 cm from the anal verge, which could be traversed with a standard gastroscope (Fig. 2). Beyond the stenosis, a recess with 2 draining orifices and abundant purulent discharge was identified. A septotomy was performed with an ITknife (Olympus America, Center Valley, Pa, USA), granting access to a 10-cm cavity containing hematopurulent material. Attempts to place an overtube for EVT were unsuccessful due to the distance from the anal verge, and modified vacuum therapy with a nasogastric tube was not feasible.

**Figure 2:**
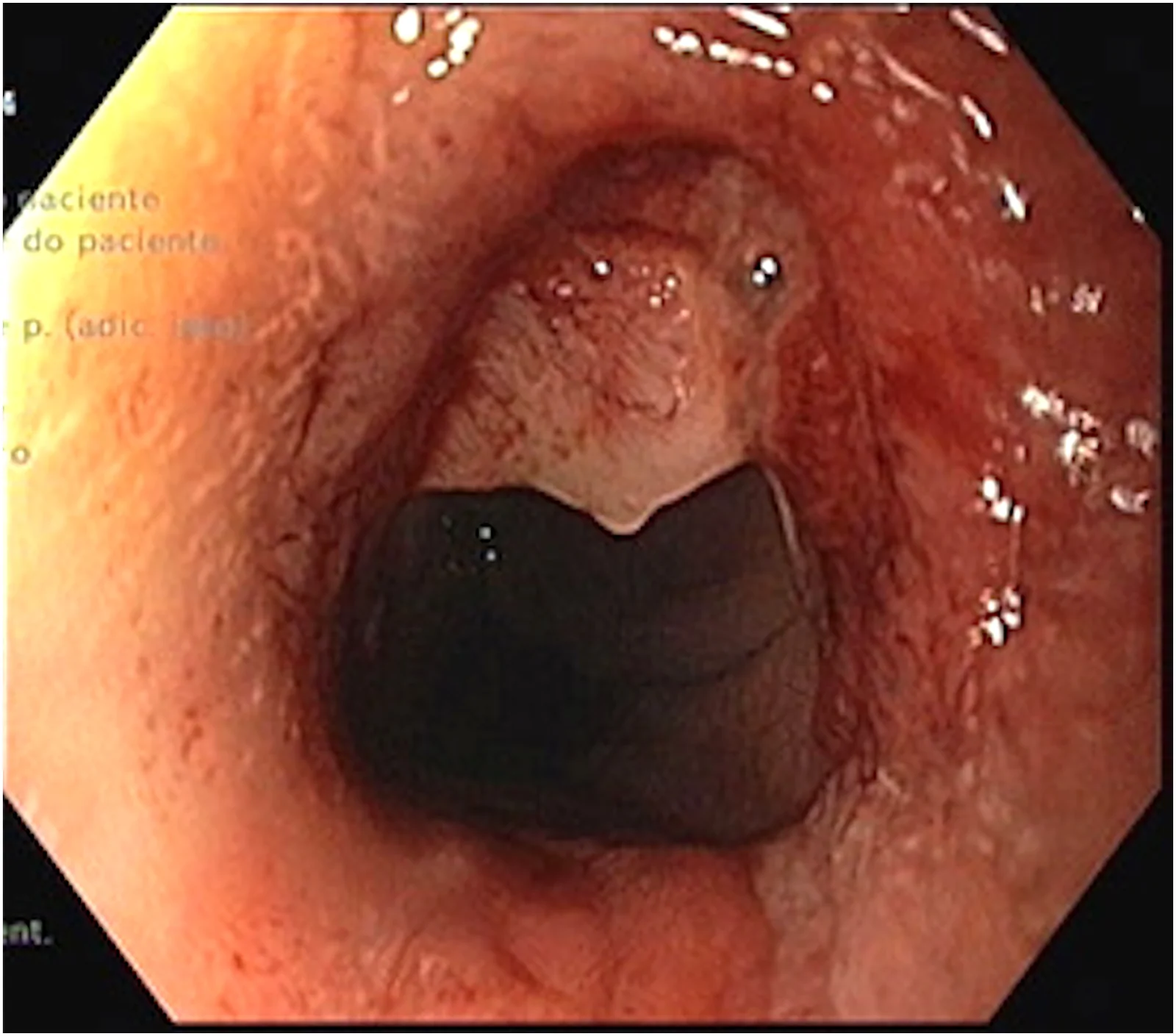
Endoscopic view of the stenosis before intervention.

On day 6 of hospitalization, we proceeded with a second endoscopic session under fluoroscopic guidance. Following careful balloon dilation of the stenosis up to 12 mm (CRE Wireguided Balloon Dilation Catheter, Boston Scientific, Marlborough, Mass, USA), 2 double-pigtail plastic stents (7F/5 cm and 7F/7 cm, Boston Scientific) were placed into the cavity (Fig. 3), with distal ends positioned below the stenosis to allow for internal drainage (Fig. 4). On day 9, a third endoscopy was performed revealing a stenotic anastomosis with minimal purulent discharge and well-positioned pigtails within the collection. Further dilation to 18 mm, alongside the pigtails, was performed, resulting in a superficial mucosal laceration at the end of the procedure. Enteral feeding was resumed at this stage. Radiologic evaluation 1 week after the initial drainage showed marked reduction of the collection to 28 × 18 mm with both double pigtails in situ. Antibiotic therapy was discontinued after 12 days, and the patient was discharged home after 16 days of hospitalization.

**Figure 3:**
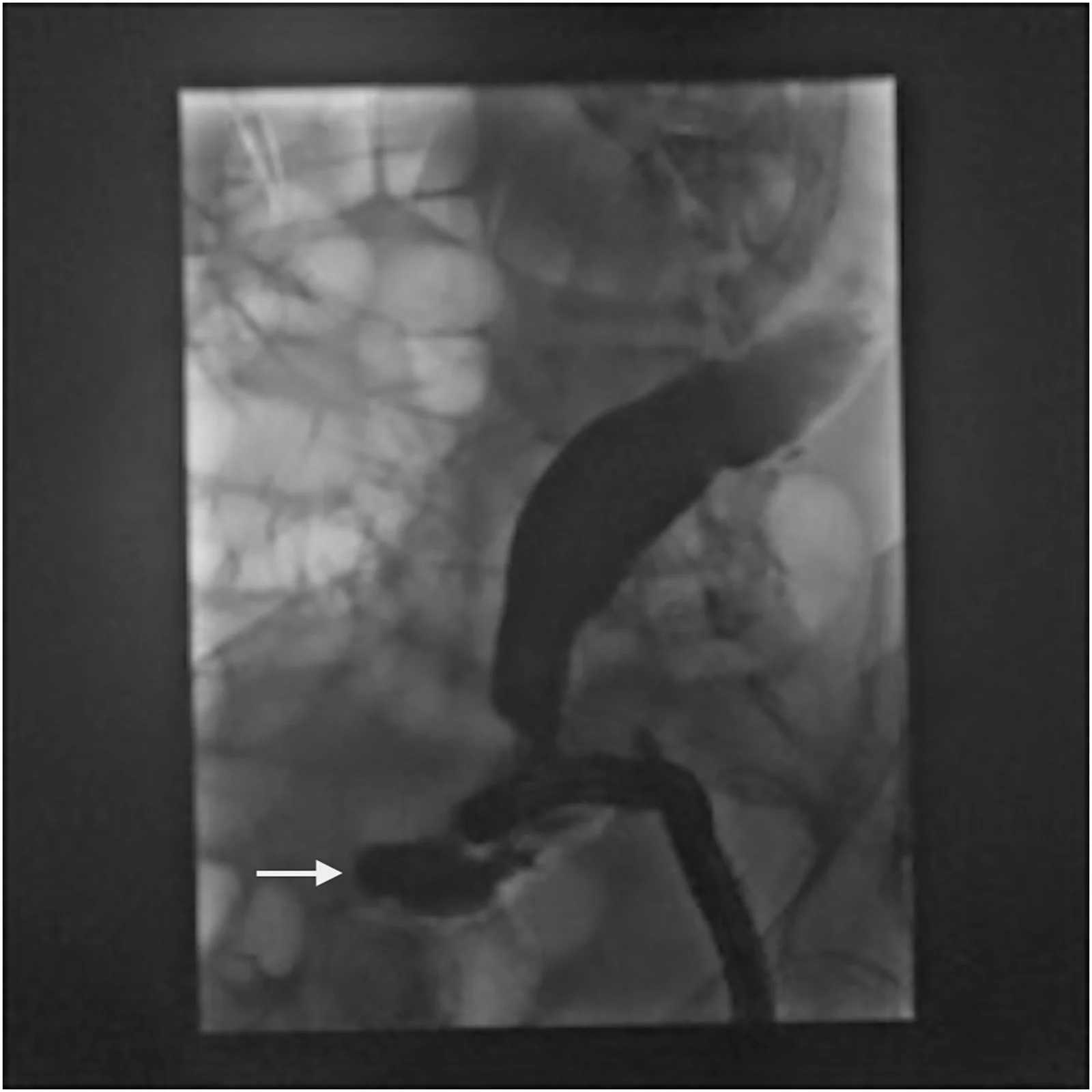
Fluoroscopic view of the perianastomotic collection (*arrow*) after contrast instillation through the endoscope (below) with partial opacification of the proximal colon (above).

**Figure 4:**
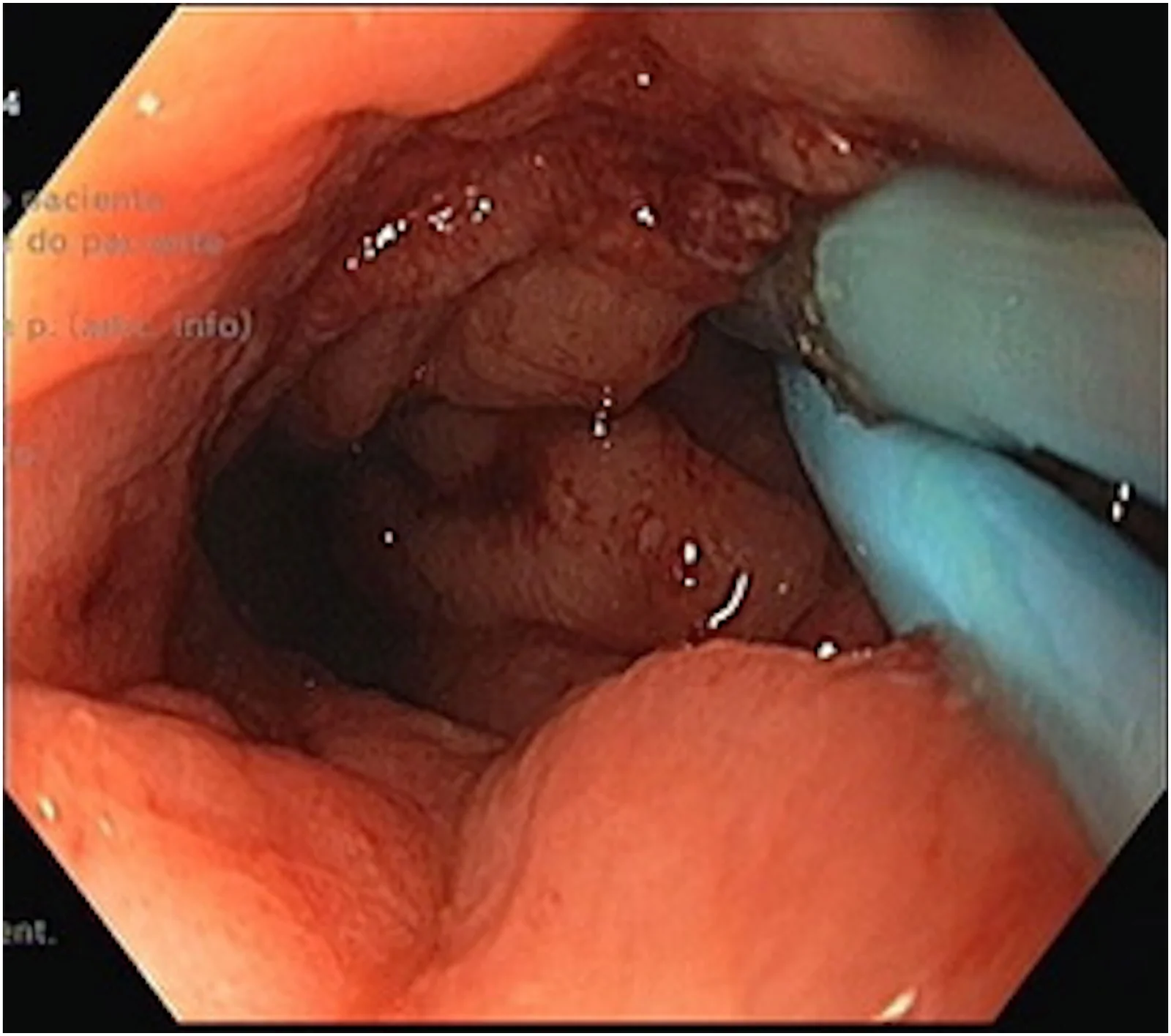
Two double-pigtail plastic stents in situ for internal drainage, viewed at the entrance of the cavity.

In the outpatient setting, over the following 2 months, she underwent 5 sessions of progressive endoscopic dilation, achieving a final diameter of 20 mm, with spontaneous migration of the plastic stents identified in the fourth session. All endoscopic procedures were performed with the patient under conscious sedation, with the patient on left-side decubitus and after a complete bowel preparation. No intraprocedure or postprocedure adverse events were reported. Either a therapeutic gastroscope (GIF-2TH180, Olympus America) or a standard gastroscope (GIF-H190N, Olympus America) was used. At the 3-month follow-up, she was asymptomatic. Endoscopic reevaluation, 4 months after initial admission, demonstrated a 15-mm anastomosis, easily traversable, with no residual fistulous orifice or purulent drainage (Fig. 5), confirmed by abdominal computed tomography (Fig. 6).

**Figure 5:**
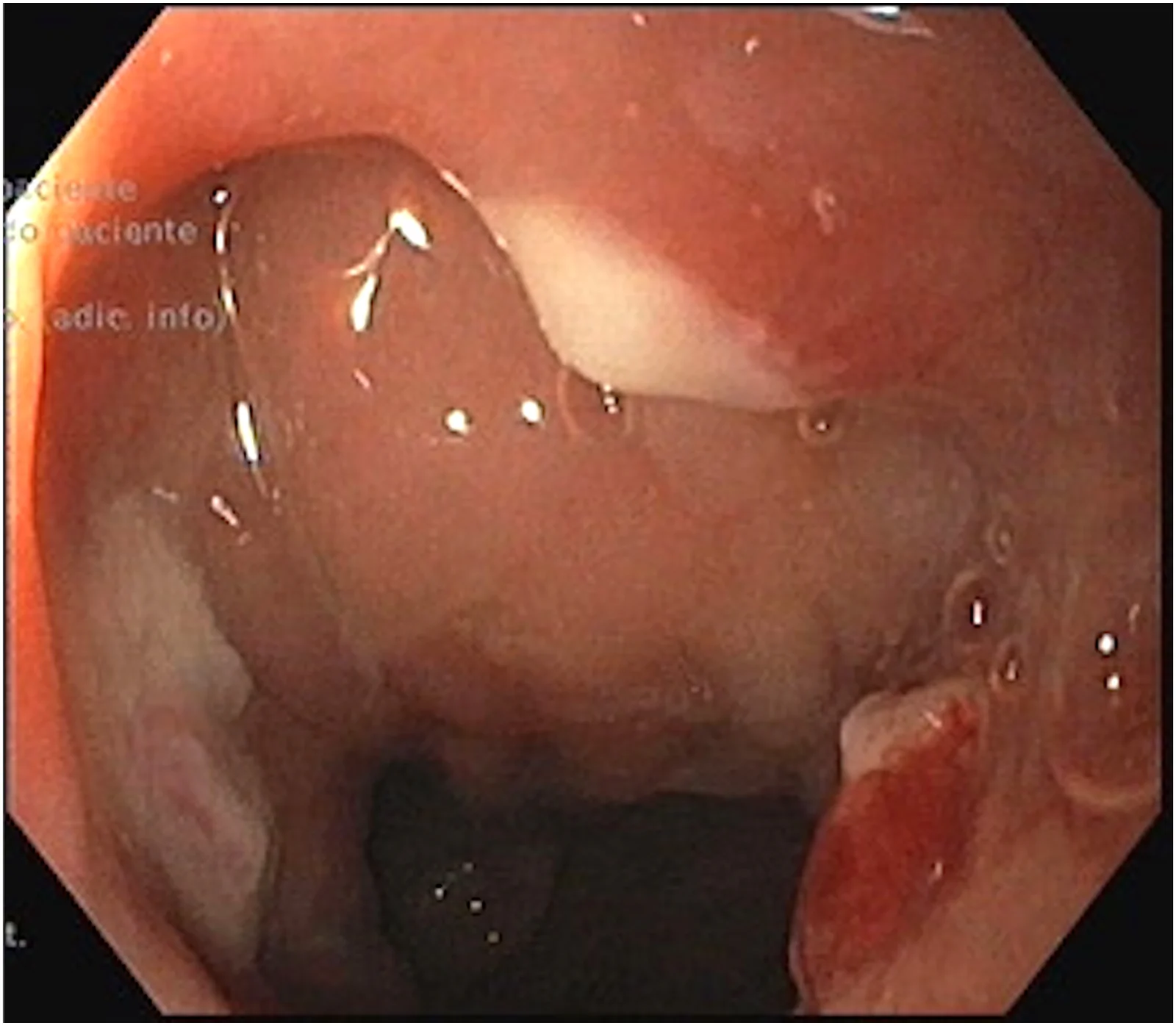
Endoscopic view of the stenosis at final follow-up.

**Figure 6:**
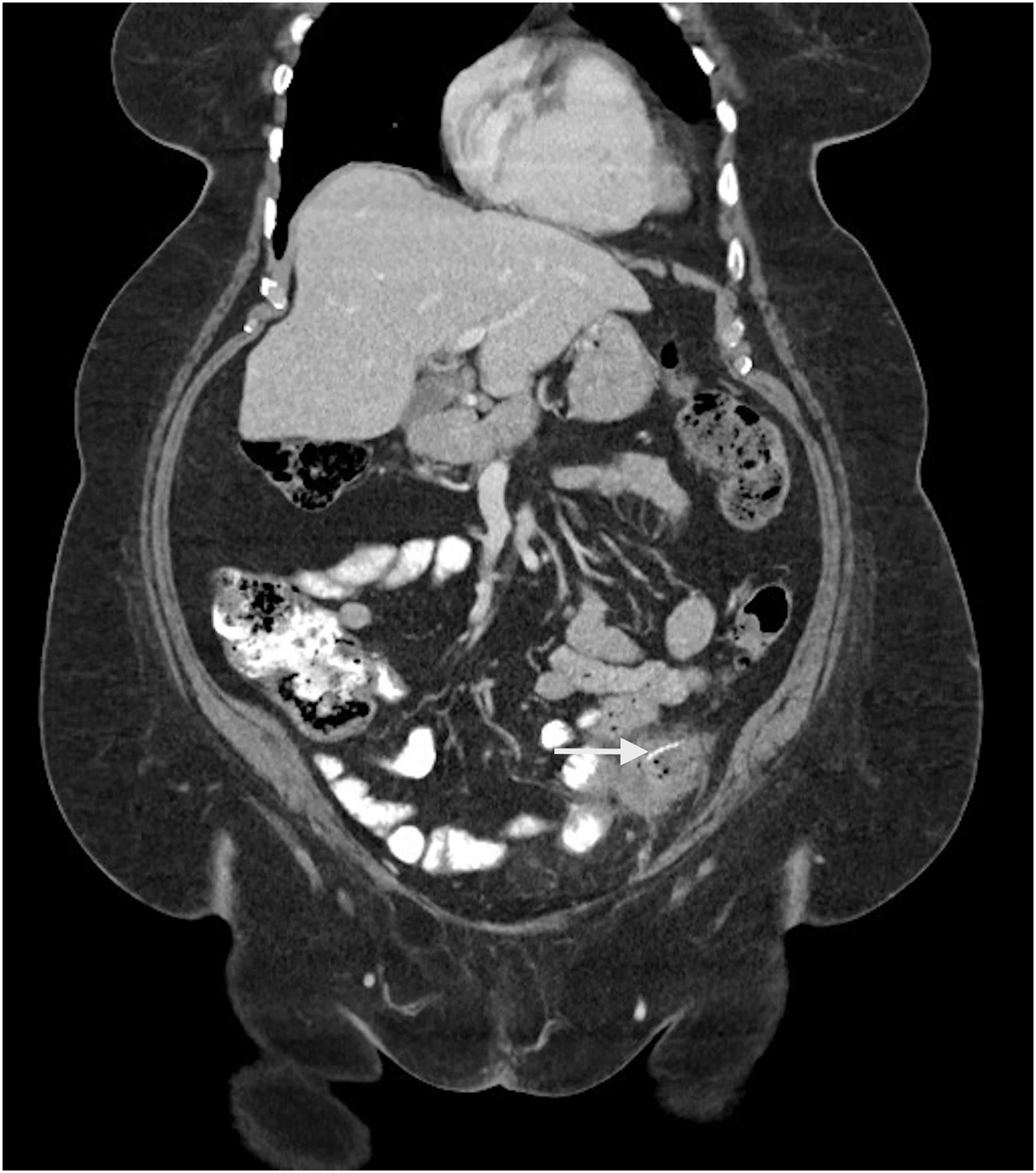
Follow-up computed tomography scan with surgical anastomosis (*arrow*).

## Discussion

Endoscopic management has emerged as a less-invasive yet effective alternative for selected cases of postoperative gastrointestinal leaks and dehiscence.

In our case, the use of double-pigtail stents allowed for continuous internal drainage of the perianastomotic collection, facilitating cavity collapse and healing, avoiding percutaneous drainage or surgical reintervention. Simultaneously, addressing the anastomotic stenosis through balloon dilation optimized drainage through reduction of bowel pressure.

From the patient's perspective, the endoscopic approach was perceived as highly satisfactory. Despite requiring multiple endoscopic procedures and repeated bowel preparations, the treatment was largely conducted in an outpatient setting after initial stabilization and allowed avoidance of surgical reintervention, stoma creation, or prolonged hospitalization. The patient reported good tolerance of the procedures and expressed preference for this minimally invasive approach over surgical alternatives, valuing the preservation of bowel continuity and faster functional recovery.

This case demonstrates the use of a minimally invasive endoscopic treatment for a challenging anastomotic dehiscence with associated stenosis, highlighting the feasibility, safety, and effectiveness of combining internal drainage and endoscopic dilation. However, several limitations of this technique should be acknowledged. The approach requires multiple endoscopic sessions, with repeated bowel preparations, which may impact patient comfort and adherence. Progressive balloon dilation of a recent anastomosis carries a potential risk of mucosal injury, bleeding, or perforation, particularly in the setting of inflammation/infection. Additionally, successful implementation depends on advanced endoscopic expertise, availability of fluoroscopic guidance, and careful patient selection, limiting its widespread applicability. Finally, the optimal timing, the number of procedures, and the duration of stent placement remain undefined. Nonetheless, our experience supports the growing role of advanced endoscopic techniques as first-line options in selected cases of colocolonic anastomotic dehiscence.

## Patient consent

Patient consent was obtained for the publication of this article.

## Disclosure

All authors disclosed no financial relationships.
